# Supplementary material for: Smooth Interpolating Curves with Local Control and Monotone Alternating Curvature
Source: Comput Graph Forum. 2022 Oct 6;41(5):25–38. doi: 10.1111/cgf.14600 (PMC9827861; doi:10.1111/cgf.14600)
Supplement: Supplementary file 1 — Supplement Material [file CGF-41-25-s001.zip › Local-Smooth-Interpolating-MonoCurvature/extern/clothoids/docs/Matlab_manual.html]

Matlab Interface Manual — Clothoids v2.0.9

### Navigation

- index
- toc
- next
- previous
- Clothoids »
- Matlab Interface Manual

# Matlab Interface Manual¶

The interface is Object Oriented and you find the following
objects in the library:

- LineSegment
- CircleArc
- Biarc
- ClothoidCurve
- PolyLine
- ClothoidList
- ClothoidSplineG2
- Triangle2D
- FresnelCS

## Installation mex/MATLAB interface¶

In directory src\_mex you find the C++ implementation of
the proposed algorithm with mex interface.
The easy way to build mexx/MATLAB interface is to download
the compiled Toolbox at
https://github.com/ebertolazzi/Clothoids/releases
and install it.
After installation in Matlab run the command `CompileClothoidsLib`.

If you want to compile the Toolbox by yourself

```
cd toolbox
ruby populate_toolbox.rb
```

run Matlab and from the comand windows of MATLAB:

```
cd toolbox
setup
CompileClothoidsLib
open('../Clothoids.prj')
```

then compile toolbox and install.

## LineSegment¶

The class `LineSegment` store a straight segment.

## Constuctors¶

```
% build segment given initial position, angle and length
L1 = LineSegment( x0, y0, theta0, L );

% build segment given initial and final point
L2 = LineSegment( [x0, y0], [x1, y1] );

% re-build segment given initial position, angle and length
L1.build( x0, y0, theta0, L );

% re-build segment given initial and final point
L2.build( [x0, y0], [x1, y1] );
```

## Methods¶

```
% translate a segment by `[tx,ty]`
L1.translate( tx, ty );

% rotate a segment by angle `ang` around center `[cx,cy]`
L1.rotate( ang, cx, cy );

% change the orgin of a segment to `[ox,oy]`
L1.changeOrigin( ox, oy );

% cut or extend the segment at curvilinear coordinates `smin` and `smax`
L1.trim( smin, smax );

% return a structure containing description of the object as a MATLAB NURBS
bs = L1.to_nurbs();

% initial and final coordinates
x = L1.xBegin(); x = L1.xEnd();
y = L1.yBegin(); y = L1.yEnd();

% segment angle direction
ang = L1.theta();

% segment length
L = L1.length();

% segment initial and final point
[p1,p2] = L1.points();

% distance point `[x,y]` to the segment
% d = distance, s = parameter of the point of segment at minimum distance
[d,s] = L1.distance(x,y);

% print descrition of the segment
L1.info();

% plot the segment
L1.plot();
% some options may be passed to plot command
L1.plot('Color','red');
```

## Evaluation Methods¶

```
% evaluate the segment at curvilinear coordinate `s` (may be a vector)
[X,Y] = L1.eval( s ); % return separate vector for X and Y coodinates
XY    = L1.eval( s ); % return 2xN matrix, N = length(s)

% first derivative respect to s
[X_D,Y_D] = L1.eval_D( s );
XY_D      = L1.eval_D( s );

% second derivative respect to s (alwais 0)
[X_DD,Y_DD] = L1.eval_DD( s );
XY_DD       = L1.eval_DD( s );

% third derivative respect to s (alwais 0)
[X_DDD,Y_DDD] = L1.eval_DDD( s );
XY_DDD        = L1.eval_DDD( s );
```

## CircleArc¶

The class `CircleArc` store an arc of a circle.

## Constuctors¶

```
% build a circle arc given initial position, angle, curvature and length
C = CircleArc( x0, y0, theta0, k, L );

% build an empty circle
C = CircleArc();

% re-build circle arc given initial position, angle, curvature and length
C.build( x0, y0, theta0, k, L );

% re-build circle arc given initial position, angle and final position
C.build_G1( x0, y0, theta0, x1, y1 );
C.build_G1( [x0, y0], theta0, [x1, y1] );

% re-build circle arc passing by 3 points
C.build_3P( x0, y0, x1, y1, x2, y2 );
C.build_3P( [x0, y0], [x1, y1], [x2, y2]  );
```

## Methods¶

```
% translate a circle arc by `[tx,ty]`
C.translate( tx, ty );

% rotate a circle arc by angle `ang` around center `[cx,cy]`
C.rotate( ang, cx, cy );

% change the orgin of a circle arc to `[ox,oy]`
C.changeOrigin( ox, oy );

% cut or extend the circle arc at curvilinear coordinates `smin` and `smax`
C.trim( smin, smax );

% scale the circle arc by `fact` factor
C.scale( fact );

% return a structure containing description of the object as a MATLAB NURBS
bs = C.to_nurbs();

% initial and final coordinates
x = C.xBegin(); x = C.xEnd();
y = C.yBegin(); y = C.yEnd();

% intial and final angle
ang = C.thetaBegin(); ang = C.thetaEnd();

% circle arc length
L = C.length();

% circle curvature
kappa = C.kappa();

% print descrition of the segment
C.info();

% plot the circle arc
C.plot();
% some options may be passed to plot command
% npts = number of points unsed to plot the circle
C.plot(npts,'Color','red');
```

## Distance Methods¶

```
% return the bounding box triangle
[p1,p2,p3] = C.bbTriangle( fact );

% distance point `[x,y]` to the circle arc
% d = distance, s = parameter of the point of segment at minimum distance
[d,s] = C.distance(x,y);
```

## Evaluation Methods¶

```
% evaluate the circle arc at curvilinear coordinate `s` (may be a vector)
[X,Y] = L1.eval( s ); % return separate vector for X and Y coodinates
XY    = L1.eval( s ); % return 2xN matrix, N = length(s)

% first derivative respect to s
[X_D,Y_D] = L1.eval_D( s );
XY_D      = L1.eval_D( s );

% second derivative respect to s
[X_DD,Y_DD] = L1.eval_DD( s );
XY_DD       = L1.eval_DD( s );

% third derivative respect to s
[X_DDD,Y_DDD] = L1.eval_DDD( s );
XY_DDD        = L1.eval_DDD( s );
```

## Biarc¶

The class `Biarc` store a biarc or two arc
connected with G1 continuity.

## Constuctors¶

```
% build a biarc fitting intial `[x0,y0]` and final `[x1,y1]` with
% assigned initial `theta0` and final `theta0` angle.
B = Biarc( x0, y0, theta0, x1, y1, theta1 );

% build an empty biarc
B = Biarc();

% re-build a biarc
B.build( x0, y0, theta0, x1, y1, theta1);

% re-build a biarc circle arc passing by 3 points
% That minimize the weighted sum of the curvature
B.build_3P( x0, y0, x1, y1, x2, y2 );
B.build_3P( [x0, y0], [x1, y1], [x2, y2]  );
```

## Methods¶

```
% translate a biarc by `[tx,ty]`
B.translate( tx, ty );

% rotate a biarc by angle `ang` around center `[cx,cy]`
B.rotate( ang, cx, cy );

% change the orgin of the biarc to `[ox,oy]`
B.changeOrigin( ox, oy );

% scale the biarc by `fact` factor
B.scale( fact );

% reverse curvilinear coordinate of the biarc
B.reverse( fact );

% return the circle arcs forming the biarc
[C1,C2] = B.getCircles();

% return a structure containing description of the object as a MATLAB NURBS
bs = B.to_nurbs();

% initial and final coordinates of first circle
x = B.xBegin0(); x = B.xEnd0();
y = B.yBegin0(); y = B.yEnd0();

% initial and final coordinates of second circle
x = B.xBegin1(); x = B.xEnd1();
y = B.yBegin1(); y = B.yEnd1();

% intial and final angle of first circle
ang = B.thetaBegin0(); ang = B.thetaEnd0();

% intial and final angle of second circle
ang = B.thetaBegin1(); ang = B.thetaEnd1();

% circle arc length for first and second circle
L0 = B.length0(); L1 = B.length0();
% total length of biarc
L = B.length();

% circle curvature for first and second circle
kappa0 = B.kappa0(); kappa1 = B.kappa1();

% print descrition of the segment
B.info();

% plot the circle arc
B.plot();
% some options may be passed to plot command
% npts   = number of points unsed to plot the circle
% fmt1/2 = cell array with formatting command for first and second cicle
npts = 100;
fmt1 = {'Color','red'};
fmt2 = {'Color','blue'};
B.plot(npts,fmt1,fmt2);
```

## Distance Methods¶

```
% distance point `[x,y]` to the biarc
% d = distance, s = parameter of the point of segment at minimum distance
[d,s] = B.distance(x,y);

% closest point to `[x,y]` onto the biarc
% d = distance, s = parameter of the point of segment at minimum distance
[x,y,s,d] = B.closestPoint(x,y);
```

## Evaluation Methods¶

```
% evaluate the biarc at curvilinear coordinate `s` (may be a vector)
[X,Y]             = B.evaluate( s ); % return separate vector for X and Y coodinates
[X,Y,theta,kappa] = B.evaluate( s ); % return also angle and curvature

% first derivative respect to s
[X_D,Y_D] = B.eval_D( s );
XY_D      = B.eval_D( s );

% second derivative respect to s
[X_DD,Y_DD] = B.eval_DD( s );
XY_DD       = B.eval_DD( s );

% third derivative respect to s
[X_DDD,Y_DDD] = B.eval_DDD( s );
XY_DDD        = B.eval_DDD( s );
```

## ClothoidCurve¶

The class `ClothoidCurve` store a clothoid curve arc.
A clothoid curve is curve where curvature change linearly
respect to the curvilinear abscissa.
The circle arc and the line segment are particular
case when curvature is constant and 0.

The G1 fitting problem of method `build_G1` implements
the algorithm described in reference [1].
Given two points and two direction associated with the points,
a clothoid, i.e. a curve with linear varying curvature is computed
in such a way it pass to the points with the prescribed direction.
The solution in general is not unique but chosing the one for
which the angle direction variation is less than `2*pi`
the solution is unique.

## Constuctors¶

```
% build a clothoid curve starting at `[x0,y0]` with angle `theta0` and curvature
% `kappa0` the curvature derivative is `dk` while `L` is the curve length.
CL = ClothoidCurve( x0, y0, theta0, kappa0, dk, L );

% build an empty clothoid curve
CL = ClothoidCurve();

% re-build a clothoid curve
CL.build( x0, y0, theta0, kappa0, dk, L );

% re-build a clothoid passing from point `[x0,y0]` with angle `theta0`
% to `[x1,y1]` with angle `theta1` (G1 fitting problem)
CL.build_G1( x0, y0, theta0, x1, y1, theta1);
```

## Methods¶

```
% translate a clothoid curve by `[tx,ty]`
CL.translate( tx, ty );

% rotate a clothoid curve by angle `ang` around center `[cx,cy]`
CL.rotate( ang, cx, cy );

% change the orgin of the clothoid curve to `[ox,oy]`
CL.changeOrigin( ox, oy );

% scale the clothoid curve by `fact` factor
CL.scale( fact );

% cut or extend the clothoid curve at curvilinear coordinates `smin` and `smax`
CL.trim( smin, smax );

% reverse curvilinear coordinate of the clothoid curve
CL.reverse( fact );

% return a structure containing description of the object as a MATLAB NURBS
bs = CL.to_nurbs();

% initial and final coordinates of the clothoid curve
x = CL.xBegin(); x = CL.xEnd();
y = CL.yBegin(); y = CL.yEnd();

% intial and final angle of the clothoid curve
ang = CL.thetaBegin(); ang = CL.thetaEnd();

% intial and final curvature of the clothoid curve
k0 = CL.kappaBegin(); ang = CL.kappaEnd();

% derivative of curvature of the clothoid curve
dk = CL.kappa_D();

% length of the clothoid curve
L = CL.length();

% point at infinity of the clothoid
% `[xp,yp]` point at curvilinear coordinate +infinity
% `[xm,ym]` point at curvilinear coordinate -infinity
[xp,yp,xm,ym] = CL.infinity();

% print descrition of the clothoid curve
CL.info();

% return the parameters defining the clothoid curve
[x0,y0,theta0,k0,dk,L] = CL.getPars();

% plot the clothoid curve
CL.plot();
% some options may be passed to plot command
% npts = number of points unsed to plot the circle
npts = 100;
CL.plot(npts,'Color','red');
```

## Distance and intersection Methods¶

```
% distance point `[x,y]` to the clothoid curve
% d = distance, s = parameter of the point of segment at minimum distance
[d,s] = CL.distance(x,y);

% closest point to `[x,y]` onto the clothoid curve
% d = distance, s = parameter of the point of segment at minimum distance
[x,y,s,d] = CL.closestPoint(x,y);

% compute all the intersection of curve CL with curve CL1.
% CL1 may be a ClothoidCurve a CircleArc or a LineSegment object
% s  = vector of curvilinear coordinates on CL of the intersection
% s1 = vector of curvilinear coordinates on CL1 of the intersection
[s,s1] = CL.intersect(CL1);
```

## Evaluation Methods¶

```
% evaluate the clothoid curve at curvilinear coordinate `s` (may be a vector)
[X,Y]             = CL.evaluate( s ); % return separate vector for X and Y coodinates
[X,Y,theta,kappa] = CL.evaluate( s ); % return also angle and curvature

% first derivative respect to s
[X_D,Y_D] = CL.eval_D( s );
XY_D      = CL.eval_D( s );

% second derivative respect to s
[X_DD,Y_DD] = CL.eval_DD( s );
XY_DD       = CL.eval_DD( s );

% third derivative respect to s
[X_DDD,Y_DDD] = CL.eval_DDD( s );
XY_DDD        = CL.eval_DDD( s );
```

an offset respect to the normal of the curve can be used

```
% evaluate the clothoid curve at curvilinear coordinate `s` (may be a vector)
[X,Y]             = CL.evaluate( s, offs ); % return separate vector for X and Y coodinates
[X,Y,theta,kappa] = CL.evaluate( s, offs ); % return also angle and curvature

% first derivative respect to s
[X_D,Y_D] = CL.eval_D( s, offs );
XY_D      = CL.eval_D( s, offs );

% second derivative respect to s
[X_DD,Y_DD] = CL.eval_DD( s, offs );
XY_DD       = CL.eval_DD( s, offs );

% third derivative respect to s
[X_DDD,Y_DDD] = CL.eval_DDD( s, offs );
XY_DDD        = CL.eval_DDD( s, offs );
```

## ClothoidList¶

Store a list of clothoids to be used as a single spline.

Documentation will be available soon, see examples in
`tests` for the moments

## ClothoidSplineG2¶

Implements the algorithm described in references [2] and [3].
documentation will be available soon, see examples in
`tests` for the moments

## Triangle2D¶

## FresnelCS¶

### Authors¶

- Enrico Bertolazzi

  Dipartimento di Ingegneria Industriale

  Università degli Studi di Trento

  email:

  homepage: https://e.bertolazzi.dii.unitn.it
- Marco Frego

  Faculty of Science and Technology

  Free University of Bozen-Bolzano

  email:

### References¶

1. *E. Bertolazzi, M. Frego*, **G1 fitting with clothoids**,

   Mathematical Methods in the Applied Sciences,

   John Wiley & Sons, 2014, vol. 38, n.5, pp. 881-897,

   https://doi.org/10.1002/mma.3114
2. *E. Bertolazzi, M. Frego*, **On the G2 Hermite interpolation
   problem with clothoids**,

   Journal of Computational and Applied Mathematics,

   2018, vol. 15, n.341, pp. 99-116.

   https://doi.org/10.1016/j.cam.2018.03.029
3. *E. Bertolazzi, M. Frego*, **Interpolating clothoid splines with
   curvature continuity**,

   Mathematical Methods in the Applied Sciences,

   2018, vol. 41, n.4, pp. 1099-1476.

   https://doi.org/10.1002/mma.4700
4. *E. Bertolazzi, M. Frego* **Point-Clothoid Distance and Projection Computation**,

   SIAM Journal on Scientific Computing,

   2019, 41(5), A3326–A3353.

   https://doi.org/10.1137/18M1200439
5. *E. Bertolazzi, M. Frego* **A Note on Robust Biarc Computation**,

   Computer-Aided Design & Applications 16 (5), 822-835

   http://www.cad-journal.net/files/vol\_16/CAD\_16(5)\_2019\_822-835.pdf

### Quick search

### Table of Contents

- Matlab Interface Manual
  - Installation mex/MATLAB interface
  - LineSegment
  - Constuctors
  - Methods
  - Evaluation Methods
  - CircleArc
  - Constuctors
  - Methods
  - Distance Methods
  - Evaluation Methods
  - Biarc
  - Constuctors
  - Methods
  - Distance Methods
  - Evaluation Methods
  - ClothoidCurve
  - Constuctors
  - Methods
  - Distance and intersection Methods
  - Evaluation Methods
  - ClothoidList
  - ClothoidSplineG2
  - Triangle2D
  - FresnelCS
- C++ API
- MATLAB API

«
hide menu

menu
sidebar
»

### Navigation

- index
- toc
- next
- previous
- Clothoids »
- Matlab Interface Manual

© Copyright 2021, Enrico Bertolazzi and Marco Frego.
Created using Sphinx 4.2.0.
